# Prognostic characterization of immune molecular subtypes in non-small cell lung cancer to immunotherapy

**DOI:** 10.1186/s12890-021-01765-3

**Published:** 2021-11-29

**Authors:** Chenlu Li, Jingjing Pan, Jing Luo, Xupeng Chen

**Affiliations:** 1 Department of Gastroenterology, Affiliated Yueqing Hospital, Wenzhou Medical University, Wenzhou, 325600 Zhejiang People’s Republic of China; 2 Department of Laboratory Medicine, The First Affiliated Hospital of Wenzhou Medical University, Wenzhou, People’s Republic of China; 3 Department of Rheumatology, The First Affiliated Hospital of Wenzhou Medical University, Wenzhou, 325035 People’s Republic of China

**Keywords:** Non-small cell lung cancer, Immune molecular subtype, Risk stratification, Immunotherapy, Prognosis

## Abstract

**Background:**

Non-small cell lung cancer (NSCLC) was usually associated with poor prognosis and invalid therapeutical response to immunotherapy due to biological heterogeneity. It is urgent to screen reliable biomarkers, especially immunotherapy-associated biomarkers, that can predict outcomes of these patients.

**Methods:**

Gene expression profiles of 1026 NSCLC patients were collected from The Cancer Genome Atlas (TCGA) datasets with their corresponding clinical and somatic mutation data. Based on immune infiltration scores, molecular clustering classification was performed to identify immune subtypes in NSCLC. After the functional enrichment analysis of subtypes, hub genes were further screened using univariate Cox, Lasso, and multivariate Cox regression analysis, and the risk score was defined to construct the prognostic model. Other microarray data and corresponding clinical information of 603 NSCLC patients from the GEO datasets were applied to conduct random forest models for the prognosis of NSCLC with 100 runs of cross-validation. Finally, external datasets with immunotherapy and chemotherapy were further applied to explore the significance of risk-scores in clinical immunotherapy response for NSCLC patients.

**Results:**

Compared with Subtype-B, the Subtype-A, associated with better outcomes, was characterized by significantly higher stromal and immune scores, T lymphocytes infiltration scores and up-regulation of immunotherapy markers. In addition, we found and validated an eleven -gene signatures for better application of distinguishing high- and low-risk NSCLC patients and predict patients’ prognosis and therapeutical response to immunotherapy. Furthermore, combined with other clinical characteristics based on multivariate Cox regression analysis, we successfully constructed and validated a nomogram to effectively predict the survival rate of NSCLC patients. External immunotherapy and chemotherapy cohorts validated the patients with higher risk-scores exhibited significant therapeutic response and clinical benefits.

**Conclusion:**

These results demonstrated the immunological and prognostic heterogeneity within NSCLC and provided a new clinical application in predicting the prognosis and benefits of immunotherapy for the disease.

**Supplementary Information:**

The online version contains supplementary material available at 10.1186/s12890-021-01765-3.

## Background

As one of the most common tumors with high morbidity and mortality, lung cancer leads to a poor prognosis and increases critical social burden [1]. Non-small cell lung cancer (NSCLC) is the most common histological subtype with its unique biological characteristics and accounts for approximately 85% of all lung cancers [2]. More than 50% of diagnosed NSCLC patients were at advanced stages and the prognosis of NSCLC was relatively poor with only 11%-15% overall 5-year survival rate [3]. Despite remarkable progress has been made for the treatment of NSCLC, including radiotherapy, chemotherapy and surgery according to different locations and clinical stages of NSCLC, there is still lack of effective strategies for the advanced NSCLC treatment [4]. Recently, the rapid rise of immunotherapy has brought a new therapeutic landscape for the NSCLC patients who didn’t benefit from conventional chemotherapy, radiation or surgery [5]. However, in clinical practice, the majority of NSCLC patients were usually still lack effective therapeutical response to immunotherapy [6, 7]. Therefore, it is crucial to screen reliable biomarkers, especially immunotherapy-related biomarkers, that can predict outcomes of NSCLC patients.

In clinical practice, the TNM stage system was acknowledged as the most frequently used tool to predict the prognosis of NSCLC patients, which majorly depended on the inherent anatomical abnormity, including tumor size, lymph node situation and distant metastatic status [8]. However, the existence of tumor genetic and biological heterogeneity made it inaccurate for the TNM system to predict disease progression and prognosis [9]. The growing researches focused on the biological heterogeneity for the different prognoses in NSCLC. Traditionally, it's well-know that that histological subtypes of NSCLC were significantly associated with the prognosis of the tumor [4]. In addition, the application of immunohistochemical technology had also identified a series of lung adenocarcinoma (LUAD) and lung squamous cell carcinoma (LUSC) markers, such as *EGFR* and *ALK* mutation testing to refine particular subtypes of NSCLC classification [10]. Moreover, recent studies have demonstrated that genetic characteristics, including *PD-L1* associated mRNA signatures [11], immune infiltration-associated lncRNA signatures [12] and serum microRNA signatures [13], are emerging as critical prognostic elements in predicting the outcomes for NSCLC patients. Interactions between cancer cells and tumor-infiltrating immune cells in the tumor microenvironment (TME) have been reported essential to cancer progression and aggressiveness [14, 15]. In addition, there was evidence that various types of immune cells infiltrated in the TME of NSCLC were associated with different clinical outcomes [16]. Hence, identification of molecular subtypes of NSCLC based on tumor-infiltrated immune cells and prognosis-related biomarkers is increasingly recognized to facilitate personalized treatment selection and improve disease management.

This study aimed to identify clustering immune subtypes of NSCLC and systematically assess the correlation between the characteristics of subtypes and prognosis, immunotherapy, and somatic mutation in NSCLC. Combining the prognostic gene signatures and classical clinical features, the risk model was established to improve predictive risk stratification and facilitate making a treatment decision for NSCLC patients. We are convinced that our findings would help to gain a further insight into the prognostic signatures of NSCLC and provide promising strategies for NSCLC immunotherapy.

## Methods

### Data preparation

The publicly available RNA-seq transcriptome data of 1026 NSCLC patients (including 522 LUAD and 504 LUSC) were collected from The Cancer Genome Atlas (TCGA) datasets (https://portal.gdc.cancer.gov/). The level 3 gene expression profile was integrated by the Illumina HiSeq 2000 RNA Sequencing platform and normalized as the FPKM form. The corresponding clinical data and somatic mutation data of these patients were also downloaded from TCGA for subsequent analysis, including age, gender, survival status and time, pathological stages, and TNM staging. Moreover, the microarray data and corresponding clinical information of 603 NSCLC patients were downloaded from the Gene Expression Omnibus (GEO) datasets (https://www.ncbi.nlm.nih.gov/geo/) as the external test datasets, including GSE37745, GSE31210 and GSE50081. The baseline clinicopathological signatures of these cohorts were summarized in Additional file 4: Table 1. To further investigate the expression of hub genes at protein levels, the Human Protein Atlas (HPA) [17] was applied to display the results of immunohistochemical technique.

**Table 1:** Clinical information of LUAD and LUSC in TCGA datasets

Variables	LUAD (522)		LUSC (504)	
	Number	Percentage (%)	Number	Percentage (%)
Age				
< 65	223	42.72	170	33.73
≥ 65	280	53.64	325	64.48
Unknow	19	3.64	9	1.79
Gender				
Male	242	46.36	373	74.01
Female	280	53.64	131	25.99
Survival status	242	46.36		
Alive	334	63.98	286	56.75
Dead	188	36.02	218	43.25
Survival time/days	902.51 ± 891.28		968.42 ± 957.61	
Clinical stage				
Stage I–II	403	77.20	408	80.95
Stage III–IV	111	21.26	92	18.25
Unknow	8	1.53	4	0.79
T stage				
I–II	453	86.78	409	81.15
III–IV	66	12.64	95	18.85
TX	3	0.57		
N stage				
N0	335	64.18	320	63.49
NX	11	2.11	6	1.19
N1–3	175	33.52	178	35.32
Unknow	1	0.19		
M stage				
M0	353	67.62	414	82.14
M1	25	4.79	7	1.39
MX	140	26.82	79	15.67
Unknow	4	0.77	4	0.79

### Identification of immune molecular subtypes and characteristics of subtypes

The ESTIMATE algorithm was applied to calculate the immune score for each patient using the R package “estimate” [18] and the fraction of 22 immune cell types for each patient was further identified using the CIBERSORT algorithm [19] based on the RNA-seq data (https://cibersort.stanford.edu/). Furthermore, based on the immune score, the “ConsensusClusterPlus” package [20] was used with 1000 iterations and 80% resample rate to classify the LUAD and LUSC patients into different subtypes, respectively. To comprehensively elucidate the immune characteristics of these subtypes, multiple comparisons between subgroups was performed including tumor microenvironment analysis, immune checkpoint analysis, and clinical signatures comparison. Kaplan–Meier survival analysis of immune subtypes for overall survival in NSCLC was performed using R packages “survival” [21] and “survminer” [22]. In addition, comprehensive mutation analysis was conducted by R package “maftools” and mutational signatures of the top 20 genes were further chosen subsequent comparison among immune subtypes in LUAD and LUSC.

### Functional enrichment analysis

To functionally elucidate the biological characteristics of subtypes of LUAD and LUSC, the differential expression analysis was performed using the “limma” R package [23] and Gene Ontology (GO) enrichment analysis was applied to conduct functional annotation of differential genes between groups [24]. The different expression genes (DEGs) were identified according to the following criteria: adjusted p-value < 0.05 and absolute value of log2 fold change (FC) > 1. These DEGs were visualized in the volcano plots using “ggplot2” package [25] and the common DEGs exhibited by the Venn diagram using the “jvenn” online tool [26]. The Gene set enrichment analysis (GSEA) was performed in the gene set “c2.cp.kegg.v7.2.symbols.gmt” of MSigDB by using the GSEA v4.0 software with the 1,000 permutations random sampling [27]. The significant enrichment pathway was identified by utilizing the false discovery rate (FDR) < 0.05 and the normalized enrichment score (NES).

### Identification of hub genes and Risk scores

To further screen the prognosis-related genes of NSCLC, we conducted univariate Cox proportional-hazards regression analysis to preliminarily filter significant genes through using “coxph” function in “survival” R package. Subsequently, to remove the multicollinearity among these candidate genes, the LASSO regression was applied to screen independent prognosis-related genes with the optimal penalty parameter and the minimum 10-fold Cross-Validation [28]. After further adjustment, multivariate Cox regression (stepwise model) was conducted to identify hub genes, and the coefficients obtained from the regression algorithm were used to acquire the risk score based on the following formula: $\mathrm{risk score}=\mathrm{val}\left(\mathrm{Gene}1\right)*\upbeta 1+\mathrm{val}\left(\mathrm{Gene}2\right)*\upbeta 2+\dots +\mathrm{val}\left(\mathrm{Gene n}\right)*\mathrm{\beta n}$ Moreover, according to the above formula, the risk scores of NSCLC patients were separately calculated and patients were divided into high- and low-subgroups according to the median value as the cut-off value [29].

### Prognostic model construction and evaluation

To further clarify the characteristic of risk scores, we also performed multiple analyses based on high- and low- risk groups for 1001 NSCLC patients including Kaplan–Meier survival analysis, immune checkpoint analysis, clinical signatures comparison and immune infiltration analysis. Next, the multivariate Cox regression (stepwise model) was applied to construct the predictive model for NSCLC combined risk scores and other clinical features, including age, gender, immune subtypes, clinical stages, and TNM stages. Variables with p values < 0.05 were included into the Cox regression model and the nomogram was further constructed to predict the probability of one-, three- and five-year survival in NSCLC patients using “rms” package [30]. To validate the prediction capability of the nomogram, we plotted the calibration curves of the nomogram in its 3-year and 5-year survival through a bootstrapping method with 1000 resamples. Subsequently, the clinical utility of the risk score in the prognostic nomogram model was determined by the decision curve analysis (DCA) after calculating the net benefits for patients at different risk threshold probabilities [31].

### Development and Validation of the prognostic model for NSCLC

To validate the prognostic value of risk scores in NSCLC patients, we re-performed Kaplan–Meier survival analysis and drew time-dependent receiver operating characteristic (ROC) curves using “timeROC” package [32] based on external validation set from three GEO datasets. In addition, we also compared the difference of clinical signatures between high- and low-risk subgroups in the validation set. Finally, the “randomForest” package was used to conduct random forest (RF) models for the prognosis of NSCLC with 100 runs of cross-validation, which predicted the prognosis of NSCLC based on risk scores, age, gender and TNM stage. Moreover, the ggplot2 package was applied to present the mean decrease accuracy and the mean decrease Gini index to assess the impact of each variable in RF.

### Exploration of the significance of risk-scores in clinical chemotherapy and immunotherapy response

Other two independent datasets, GSE135222 and GSE126044, were used to estimate the curative response to immunotherapy, including 27 and 16 NSCLC patients receiving immunotherapy respectively. According to current clinical guidelines, some anti-tumor drugs have been recommended for NSCLC treatment including Cisplatin, Docetaxel, Etoposide, Gemcitabine, Paclitaxel, Pemetrexed, and Vinorelbine. To evaluate the therapeutic value of risk-scores in the chemotherapy treatment for NSCLC, we calculated the half maximal inhibitory concentration (IC50) value of above chemotherapeutic drugs based on Genomics of Drug Sensitivity in Cancer (GDSC) databases. Difference of IC50 value between high and low risk-score subgroups was compared using Wilcoxon test and the results were exhibited in box diagrams using the “ggpubr” package.

### Statistical analysis

All relevant statistical analyses were performed in R software (version 3.6.1, https://www.r-project.org/). The continuous and categorical variables were presented as Mean ± Standard Deviation and number (percentages) respectively. Wilcox test was used to compare continuous variables and the Kaplan–Meier method was used to plot survival curves. The two-tailed p-value less than 0.05 was considered statistical significant.

## Results

### Significant correlation of consensus clustering for immune molecular subtypes and the clinical characteristics of NSCLC patients

**Fig. 1:**
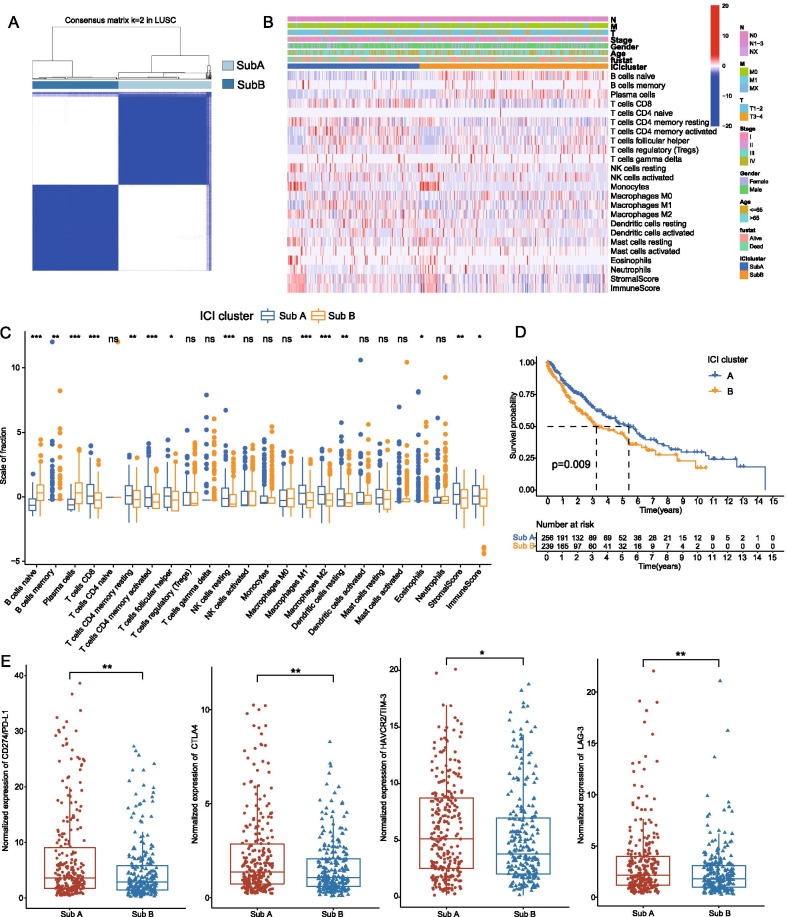
Identification of immune molecular subtypes and characteristics of subtypes in LUSC. **A** Consensus clustering matrix for k = 2 in LUSC patients. **B** Heatmap of immune cells infiltration and clinicopathologic features of the two subtypes. **C** The box plots showing the difference of immune cells infiltration between SubA and SubB. **D** Kaplan–Meier curves of overall survival (OS) for the NSCLC patients in two subtypes. **E** The expression of immune check points between SubA and SubB groups. *p < 0.05; **p < 0.01; ***p < 0.001

**Fig. 2:**
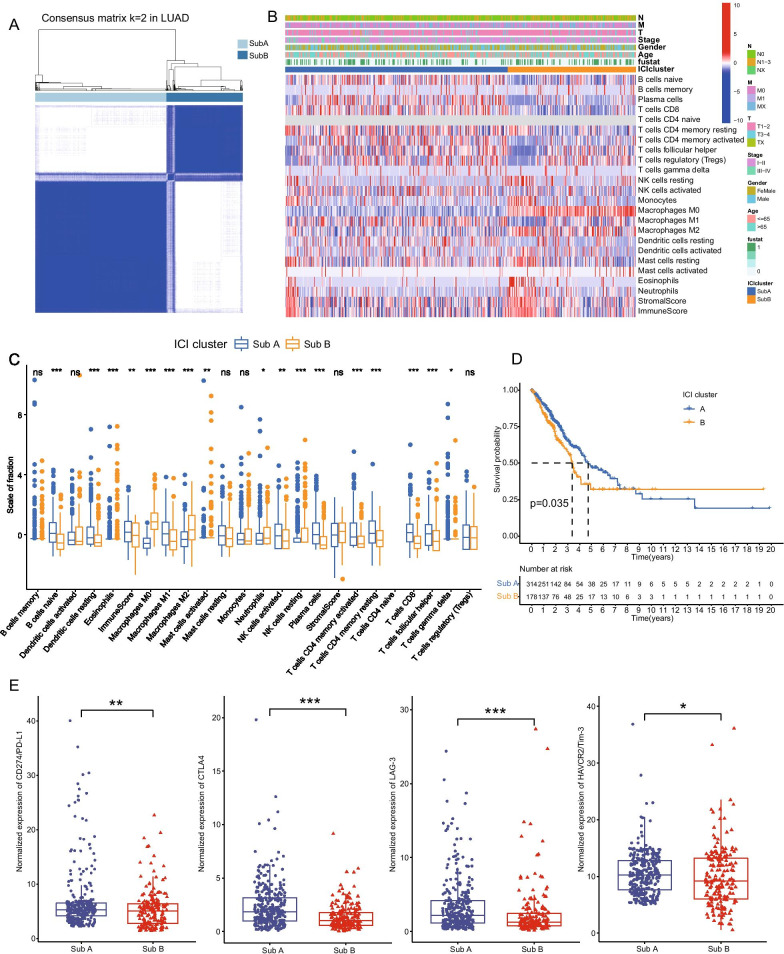
Identification of immune molecular subtypes and characteristics of subtypes in LUAD. **A** Consensus clustering matrix for k = 2 in LUAD patients. **B** Heatmap of immune cells infiltration and clinicopathologic features of the two subtypes. **C** The box plots showing the difference of immune cells infiltration between SubA and SubB. **D** Kaplan–Meier curves of overall survival (OS) for the NSCLC patients in two subtypes. **E** The expression of immune check points between SubA and SubB groups. *p < 0.05; **p < 0.01; ***p < 0.001

Additional file 1: Fig. 1 exhibited the whole workflow of our study. In this study, the RNA-seq data of 522 LUAD and 504 LUSC patients from TCGA datasets and microarray sequencing data of 603 NSCLC patients from GEO datasets were included with corresponding clinicopathological signatures. Clinicopathological characteristics of patients in the TCGA datasets and GEO datasets are shown in Table 1 and Additional file 4: Table 1, respectively. Based on the tumor-infiltrating immune scores and the percentage of fuzzy clustering measures, the k = 2 was identified as the optimum clustering model from k = 2 to k = 9 in both LUAD and LUSC groups. To further clarify the intra-patient heterogeneity of NSCLC, we distinguished these patients into two subtypes, namely, SubA (n = 261 in LUSC and 290 in LUAD) and SubB (n = 243 in LUSC and 232 in LUAD) based on the clustering immune infiltration scores (Figs. 1A, 2A). In terms of the immune infiltration scores, B lymphocytes (including naive B cells and plasma cells) were significantly increased in SubB than that of SubA. However, T lymphocytes were significantly infiltrated in SubA cohorts including memory CD4 + T cells, follicular helper T cells and CD8 + T cells. Higher stromal scores and immune scores were also detected in SubA patients than SubB groups in the tumor microenvironment of LUAD and LUSC (Figs. 1B, 2B). Subsequently, we also compared the clinicopathologic characteristics of NSCLC between the two subtypes and found that there was no significant difference in clinical features in both LUAD and LUSC patients (Additional file 3: Fig. 3A, B). Furthermore, the survival analysis showed that SubA had a longer median survival time than SubB groups regardless of LUAD or LUSC indicating the SubA patients might have a better prognosis for NSCLC (Figs. 1D, 2D). Higher expression levels of immune check points including *PD-L1, CTLA-4, LAG-3* and *TIM-3* was also validated in the SubA cohorts, suggesting those patients might be more sensitive to the immunotherapy of NSCLC (Figs. 1E, 2E).

**Fig. 3:**
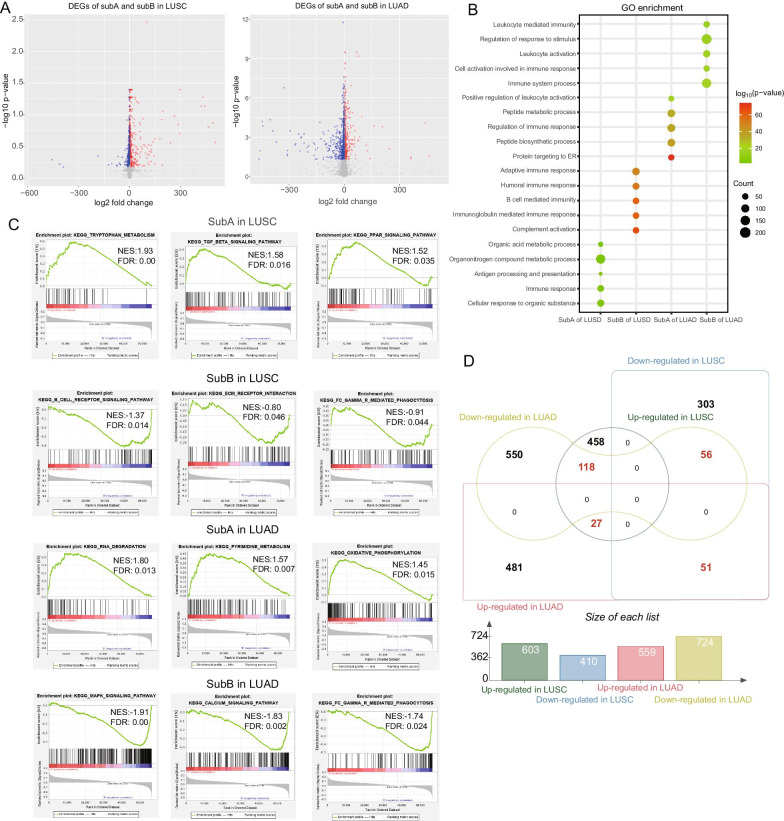
Identification of DEGs of subtypes and functional enrichment analysis. **A** Volcano plots displayed the up-regulated and down-regulated DEGs between two subgroups in LUSC and LUAD cohorts. **B** The bubble diagram showed the results of GO enrichment analysis of the subtypes. **C** The results of GSEA of SubA and SubB in LUAD and LUSC respectively. **D** Venn chart exhibited the common 252 DEGs among these subgroups in NSCLC

### Somatic variations analysis between subtypes of NSCLC

To investigate the difference of somatic variations between two subtypes in LUAD and LUSC patients, we performed mutation analysis based the corresponding somatic variations data from TCGA database. For LUSC patients, regardless of SubA and SubB, tumor samples with somatic variations occupied a high proportion in all patients (95.79% in SubA and 98.35% in SubB) and various mutation patterns were identified including missense mutation, nonsense mutation, nonstop mutation, translation start site, splice site and multi hit (Additional file 2: Fig. 2A, B). Massive mutations were also observed in LUAD patients (86.83% in SubA and 89.95% in SubB) and the types of mutations were more abundant in LUAD than LUSC cohorts including “Frame Shifts Del”, “In Frame Del” and “Frame Shift Ins” (Additional file 2: Fig. 2C, D). In addition, Additional file 2: Fig. 2 showed the top 20 genes according the rank of mutation numbers and *TTN* was the most mutable gene for LUSC patients while *TP53* was the most common on LUAD subgroups. Interestingly, there was no significant difference on the frequency of mutations between two subtypes both in LUSC and LUAD patients.

### Identification of DEGs of subtypes and functional enrichment analysis

Considering the biological characteristics of immune subtypes in NSCLC, we conducted different expression analysis between the two subtypes. Through comparing SubA with SubB groups, a total of 1013 DEGs (including 603 up-regulated and 410 down-regulated genes) in LUSC and 1283 DEGs (including 559 up-regulated and 724 down-regulated genes) in LUAD patients were identified respectively and 252 common DEGs were chosen for subsequent analysis (Fig. 3A, D). In order to further interpret biological processes and pathways of immune subtypes, these different expression genes were chosen to performed GO and GSEA analysis. It turned out that SubA cohorts were significantly enriched in immunoregulation and metabolism associated pathways such as “Positive regulation of leukocyte activation”, “Organic acid metabolic process”, and “Peptide metabolic process” while Sub B groups were enriched in B cells associated biological processes including “B cell mediated immunity”, “Immunoglobulin mediated immune response” and “Cell activation involved in immune response” (Fig. 3B). Moreover, the results of GSEA also displayed the accordant pathways for two subtypes including “TGF-β signaling pathway” “PPAR signaling pathway”, “Pyrimiding metabolism”, “oxidative phosphorylation” for SubA and “B cell receptor signaling pathways”, “MAPK signaling pathways” “Fc_Gamma_R_mediated_phagocytosis” for Sub B cohorts (Fig. 3C).

### Establishment and assessment of the risk prognosis signature

**Fig. 4:**
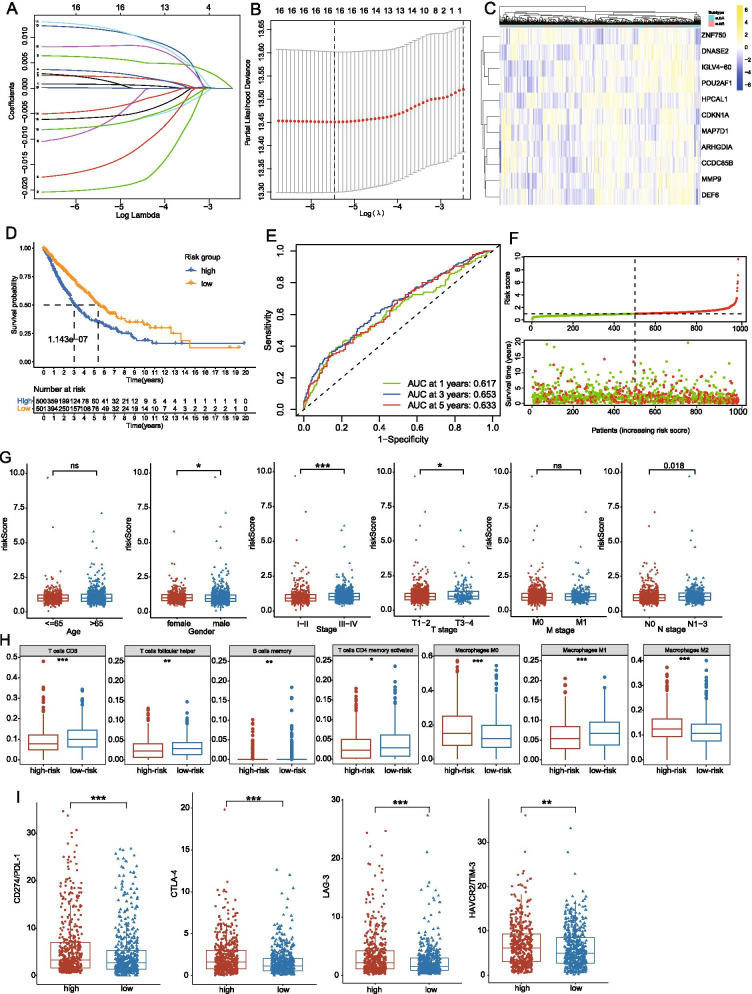
Establishment and assessment of the risk prognosis signatures through LASSO and multivariate Cox regression analysis; Correlation between risk prognosis signatures with clinical and immune characteristics. **A** LASSO coefficient profiles of 16 prognostic immune-related genes. **B** 10-times cross-validation for tuning parameter selection in the LASSO model. **C** Heatmap of the expression of 11 risk genes after multivariate Cox regression analysis. **D** Kaplan–Meier curves of overall survival (OS) for the NSCLC patients in high- and low-risk groups. **E** Time-dependent receiver operating curves of 1/3/5-years survival for NSCLC patients using risk scores. **F** The distribution of risk scores and the relationship between risk scores and survival times. **G** The different levels of risk scores between different phenotypic terms. **H** The discriminative levels of immune cells infiltration between high- and low-risk groups. **I** The distinguishing expression levels of immune check points between high- and low-risk groups in NSCLC patients. *p < 0.05; **p < 0.01; ***p < 0.001

The 252 common DEGs were included in univariate Cox, LASSO and multivariate Cox regression analysis as candidate prognosis-associated genes and eventually 11 hub genes (including *ZNF750, DNASE2, IGLV4 − 60, POU2AF1, HPCAL1, CDKN1A, MAP7D1, ARHGDIA, CCDC85B, MMP9* and *DEF6*) were identified in the risk signature based on their β coefficients (Table 2; Fig. 4C). In addition, based on the immunohistochemical data from the HPA database, the expression of these risk genes at protein levels were further validated in LUAD and LUSC patients, especially for *ARHGDIA, CDKN1A* and *CCDC85B* with high expression levels (Fig. 7A, Additional file 3: Fig. 3D). Based on the expression of these genes and their corresponding β coefficients, the risk score was defined by the following formula: Risk score = 8.59e-4*MMP9 + 2.61e-3*IGLV4-60 + 7.36e-3*CDKN1A + 4.13e-3*ARHGDIA-9e-3*ZNF750-0.011*MAP7D1-0.019*POU2AF1-0.023*DEF6 + 0.014*CCDC85B + 0.016*HPCAL1-8.8e-3*DNASE2. Subsequently, NSCLC patients were divided into the high- and low-risk subgroups with the median risk score as the cut-off value and the high-risk cohorts exhibited a worse prognosis than that of low-risk patients in the TCGA datasets (Fig. 4D). Moreover, ROC analysis showed the one-year, three-year, and five-year AUC values of the risk model were 0.617, 0.653 and 0.653, respectively in the TCGA sets (Fig. 4E) and the scatter diagram showed that the number of dead patients increased along with the increase of the risk score (Fig. 4F).

**Table 2:** Results of univariate and multivariate cox regression

Gene symbol	Univariate Cox regression			Multivariate Cox regression		
	HR	HR.95% CI	p value	HR	HR.95% CI	p value
CDKN1A	1.01	1.00–1.01	2.21E−06	1.00	1.00–1.01	7.06E−02
CAV1	1.00	1.00–1.00	1.42E−03			
HYAL1	1.02	1.01–1.04	3.78E−03			
CCDC85B	1.02	1.01–1.03	4.27E−03	1.01	1.00–1.03	7.19E−02
GPR153	1.02	1.00–1.03	7.71E−03	1.01	1.00–1.01	8.62E−05
HPCAL1	1.02	1.01–1.03	8.33E−03	0.98	0.96–1.00	3.76E−02
ATL2	0.99	0.97–1.00	1.83E−02	0.99	0.98–1.00	2.83E−02
IGLV4-60	1.00	1.00–1.00	2.04E−02			
RFX5	0.98	0.96–1.00	2.07E−02	1.02	1.00–1.03	4.17E−02
DNASE2	0.99	0.98–1.00	2.08E−02			
DEF6	0.98	0.96–1.00	2.66E−02	1.00	1.00–1.00	2.52E−04
MAP7D1	1.01	1.00–1.02	3.27E−02	0.99	0.98–1.00	1.34E−01
ZNF750	0.99	0.98–1.00	3.66E−02	1.00	1.00–1.00	7.12E−02
MMP9	1.00	1.00–1.00	3.99E−02	0.98	0.96–1.00	9.62E−02
ARHGDIA	1.00	1.00–1.01	4.19E−02			
POU2AF1	0.98	0.96–1.00	4.24E−02	0.99	0.98–1.00	1.37E−01

### Correlation between prognosis signatures with clinical and immune characteristics

To investigate the interactions between risk scores and the clinical phenotype of NSCLC, we separated NSCLC patients into different subgroups based on the phenotypic terms and found that the levels of the risk score were higher in female patients and cohorts with severe conditions than their control groups, including III-IV clinical stages, T3-T4 stages and N1-N3 stages (Fig. 4G). Furthermore, immune infiltration analysis revealed that substantial immune cells were significantly inhibited in high-risk groups including CD8 + T cells, follicular helper T (Tfh) cells, activated CD4 + memory T cells and memory B cells. In addition, the transform from macrophage1 to macrophage2 was also observed in high-risk NSCLC patients and there was no significant difference in other immune cells (Fig. 4H; Additional file 3: Fig. 3C). Moreover, higher expression levels of potential immune check points were detected in high-risk groups including *PD-L1, CTLA-4, LAG-3* and *Tim-3* (Fig. 4I). All these results showed that high risk scores were closely associated with severe manifestations, immunologic suppression and immunotherapeutic susceptibility of NSCLC, indicating that the risk signatures might serve as potential tools for the prognosis of NSCLC.

### Evaluation and validation of the prognostic model for NSCLC

**Fig. 5:**
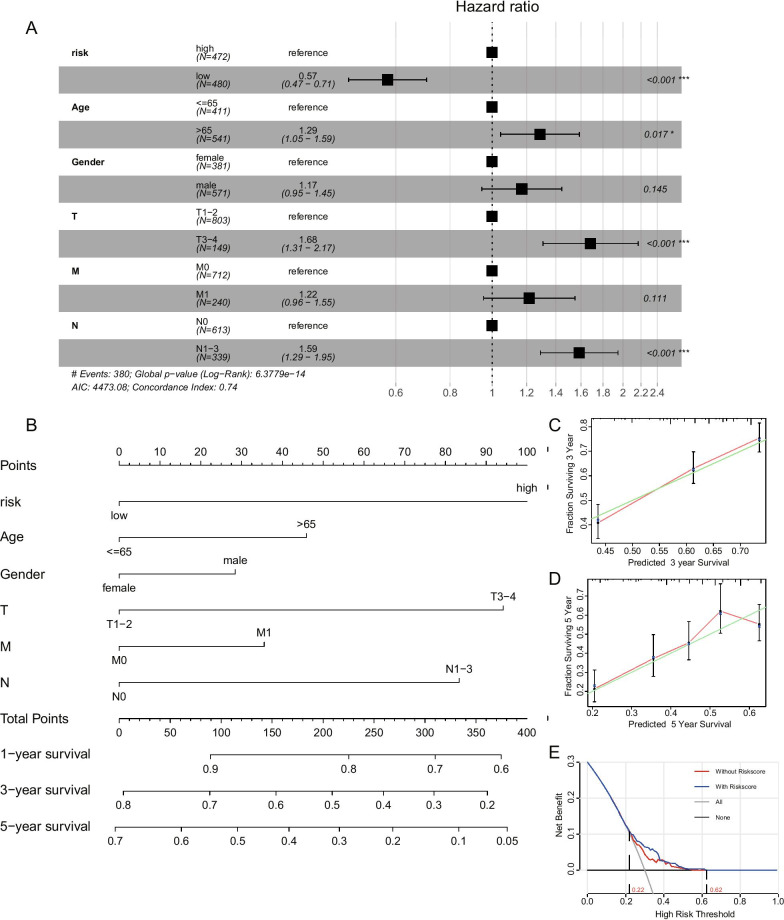
Evaluation of the prognostic model for NSCLC patients. **A** The forest plot showing the multivariable Cox model results of risk scores and other clinical features. **B** A combined nomogram for predicting the probability of 1/3/5-year survival for NSCLC patients. **C**, **D** The calibration curve of the established nomogram with 3-year and 5-year survival respectively. **F** DCA curve of the established nomogram showing risk scores could bring more benefit to the prognosis of NSCLC. *p < 0.05; **p < 0.01; ***p < 0.001

Based on the risk prognostic signatures and some primary clinical characteristics, multivariate Cox regression analysis was conducted to construct a nomogram that could accurately predict the probability of one/three/five-year survival for NSCLC patients. The risk score, age, gender and TNM stages were considered as related predictors for the prognosis of NSCLC and incorporated into the nomogram (Fig. 5A). From the nomogram, we could observe that the risk score contributed the most to the total score with the 0.74 concordance index (Fig. 5B). Calibration curves exhibited that the nomogram had a good prediction capacity in both three-year and five-year overall survival for NSCLC (Fig. 5C, D) and the clinical decision analysis showed that when the threshold probability was between 0.22 and 0.62, the net benefit of using the applied model with risk score was better than the model without risk score (Fig. 5E).

**Fig. 6:**
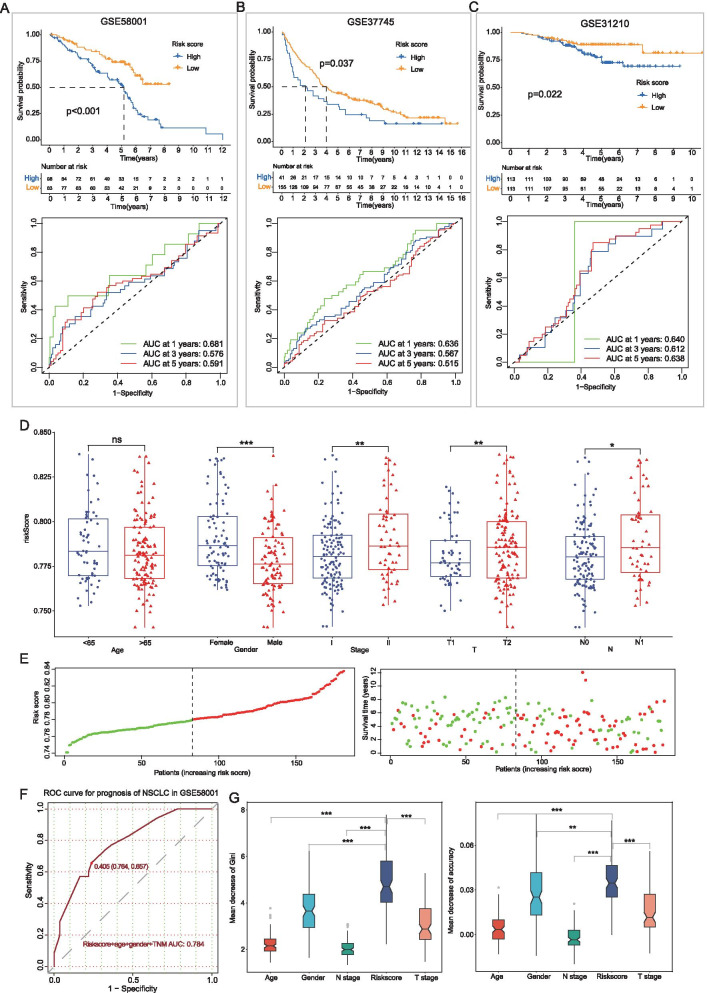
Validation of the prognostic model for NSCLC patients using external datasets. **A**–**C** Kaplan–Meier curves and ROC curves for the overall survival of NSCLC in three GEO datasets. **D** Validation of the correlation of risk scores and clinical characteristics in external datasets. **E** The distribution of risk scores and the relationship between risk scores and survival times in GEO datasets. **F** Receiver operating characteristic curve of the combined risk models for the prognosis of NSCLC with the mean AUC value 0.784. **G** Variable importance of risk scores and clinical variables of predicting the prognosis of NSCLC. Mean decrease accuracy represents the decrease of accuracy in the model when one variable is excluded, and mean decrease Gini represents the specific diagnostic capabilities of variables in the construction of the predicting model. *p < 0.05; **p < 0.01; ***p < 0.001

To further validate the predictive capacity of the risk model in external datasets, we recalculated the risk score based on the expression of 11 risk genes from three GEO datasets and performed corresponding analysis. It revealed that low-risk groups had a better prognosis and risk score could predict the overall survival for NSCLC in all datasets (one-year/three-year/five-year AUC value: 0.681/0.576/0.591 in GSE58001; 0.636/0.567/0.515 in GSE37745 and 0.640/0.612/0.638 in GSE31210) (Fig. 6A–C). In addition, higher risk scores were also found in the female and severe patients with senior TNM stages and the risk of death was also elevated with the increase of the risk score in the scatter diagram (Fig. 6D, E). Furthermore, the RF model was constructed with 100 runs of cross-validation and ROC analysis showed the risk score could be applied to predict the prognosis for NSCLC combined with age, gender and TNM stages with a high mean AUC value of 0.784 (Fig. 6F). To further evaluate the contribution of each parameter in the risk model to the prognosis of NSCLC, the RF model was assessed by ranking methods. It revealed that the risk score was the most significant index for the prognosis of NSCLC, with a higher mean decrease of Gini and Accuracy index than other clinical indexes (Fig. 6G). These results suggested the established nomogram possessed a good clinical practicability to predict the prognosis of NSCLC.

### NSCLC patients with higher risk-scores manifested better curative responses to Chemotherapy and immunotherapy

**Fig. 7:**
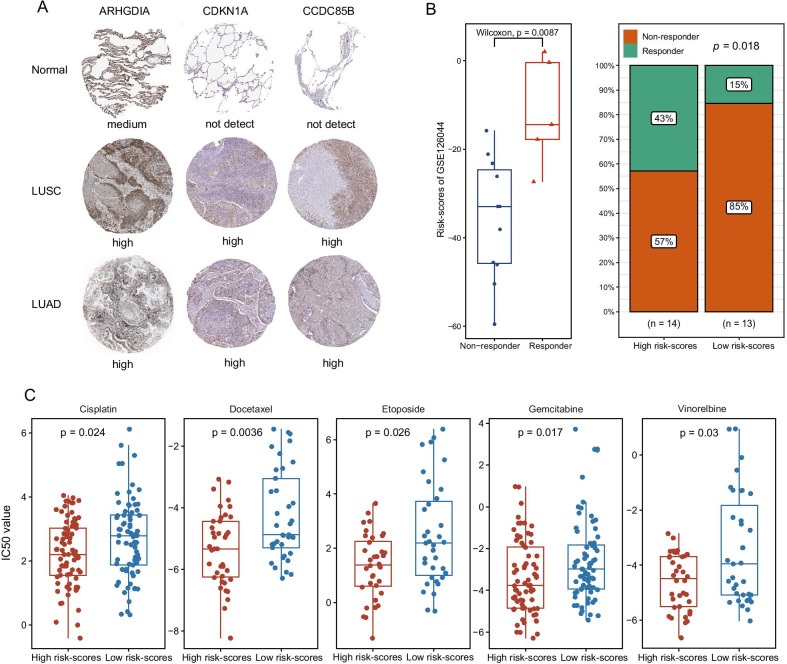
Exploration of the significance of risk-scores in clinical chemotherapy and immunotherapy response. **A** The expression of these risk genes (ARHGDIA, CDKN1A, and CCDC85B) remarkably increased in tumor patients using immunohistochemistry from HPA database. **B** the effective response rate of immunotherapy was significantly higher in the high-risk score group than in low-risk cohorts; **C** Difference of IC50 value between high- and low-risk groups for common chemotherapeutics drugs including Cisplatin, Docetaxel, Etoposide, Gemcitabine, and Vinorelbine

To further explore the role of risk-scores in predicting the therapeutic benefit in the NSCLC disease, the gene profiles of patients who accepted anti-PD-L1 immunotherapy from two GEO datasets were used to calculate risk-scores and assigned into high- and low-risk scores groups. Notably, the effective response rate of immunotherapy was significantly higher in the high-risk score group than in low-risk cohorts and the responder also exhibited higher risk-scores than non-responders (Fig. 7B). Besides immune-checkpoint blockers therapy, we also attempted to investigate the potential associations between risk-scores and the curative efficacy of common chemotherapy drugs in treating NSCLC. Interestingly, except Paclitaxel and Pemetrexed, other five drugs, including Cisplatin, Docetaxel, Etoposide, Gemcitabine, and Vinorelbine, all exhibited lower IC50 value in high risk-score groups indicating the patients with high risk-scores might obtain better curative efficacy from common chemotherapy (Fig. 7C, Additional file 3: Fig. 3E). Collectively, all these outcomes indicated that risk-scores could be regarded as a potential element associated with the response to immunotherapy and common chemotherapy in NSCLC patients.

## Discussion

As a malignant tumor with high mortality, the prognosis of NSCLC remains poor without an effective therapeutical response to immunotherapy due to tumor biological heterogeneity. In the past decade, identification of histological and molecular subtypes for NSCLC has resulted in dramatic improvements in disease outcomes [4]. Particularly, substantial molecularly targeted agents (such as *EGFR* or *ALK* inhibitors) have been approved to treat NSCLC patients with genetic alterations in corresponding protein-encoding genes [33]. However, even if the NSCLC patients were at the same clinical stage, their prognosis and therapeutical response to the same treatment might still be different in clinical practice. Skoulidis’s study has also reported this phenomenon and attributed it to genomic heterogeneity [34]. Therefore, identification of a novel subtype and reliable prognostic risk model for NSCLC is urgently needed.

In this study, we first proposed an immune molecular subtype based on clustering immune infiltration scores with distinct clinical and immunological signatures in LUAD and LUSC respectively. Interestingly, regardless of LUAD or LUSC, the characteristics of the two molecular subtypes manifested significant homogeneity. TME analysis revealed higher stromal and immune scores in SubA than that of SubB, indicating anti-tumor immune response was significantly activated in SubA of NSCLC [35]. Moreover, higher infiltration scores of T cells, especially CD8+ T cells which have been regarded as the major immune cells for anti-tumor efficacy [36], were demonstrated in the subtype A, and SubA also presented longer median survival time than SubB through Kaplan–Meier survival analysis (Figs. 2D, 3D). Immune exhaustion marker genes (such as *PD-L1, CTLA-4, LAG-3* and *HAVCR2*) have been demonstrated to play significant role in immune suppression in multiple tumors and several target inhibitors have also been widely applied to immunotherapy for cancers [37]. It was worth mentioning that the expression levels of these immune exhaustion marker genes were significantly increased in SubA subgroups suggesting a higher level of immune exhaustion and potential better therapeutical response in these tumors [38]. In addition, there was no significant difference between immune-subtypes in clinical signatures of NSCLC, implying that the current evaluation system including TNM stages failed to discriminate the molecular subtypes. Moreover, somatic mutations analysis on the frequency of mutations demonstrated no significant enrichment of mutations between two subtypes both in LUAD and LUSC, suggesting somatic mutation didn’t participate in the process of immune-subtypes in NSCLC.

To further explore the potential biological functional features of the subtypes in NSCLC, we also performed GO enrichment and GSEA analysis. Consistent with the immunological signatures of subtypes, functional enrichment analysis revealed that B cells associated biological processes including “B cell mediated immunity”, “Immunoglobulin mediated immune response” and “Cell activation involved in immune response” were more active in SubB groups while immunoregulation and amino-acid metabolism associated pathways such as “Positive regulation of leukocyte activation”, “Organic acid metabolic process” and “Peptide metabolic process” were significant enriched in the SubA cohorts. Markowitz’s study [39] has demonstrated that TGF-β signaling pathway played an important role in suppressing primary tumorigenesis in multiple tissues and Inoue et al. also found the overexpression of TGF-β was associated with the better prognosis in the 5-year survival for lung cancers [40]. In addition, cellular experiments exhibited *EGFR* inhibitors might reverse of Warburg effect, one metabolic process of the excessive conversion from glucose to lactate in cancers, and re-activate oxidative phosphorylation of cancer cells for cancer therapy [41]. Interestingly, in this study, the results of GSEA showed SubA was significantly enriched in the metabolic-related signaling pathways such as “TGF-β signaling pathway” and “oxidative phosphorylation pathway”, interpreting the better prognosis of Subtype A in NSCLC. Moreover, the SubB cohorts were also enriched in the B cell induced immune pathways such as “B cell receptor signaling pathways” and “Fc_Gamma_R_mediated_phagocytosis”, consisted with the results of GO analysis.

Furthermore, to better clarify the prognostic value of DEGs for NSCLC, we successfully screened 11 prognostic risk signatures based on LASSO regression analysis and univariate/multivariate Cox regression analysis. High-expression of these risk signatures at protein levels was confirmed by immunohistochemistry from the HPA database. Notably, based on the expression of these genes, risk scores were further identified, which effectively stratified the NSCLC patients into high- and low-risk groups in TCGA and GEO dataset respectively. Survival analysis revealed that low-risk groups had longer overall survival than patients with high riskscores and ROC curves exhibited the certain predictive capacity of risk scores for the one/three/five years survival of NSCLC. Female had been recognized as the major cohorts for the never-smokers with NSCLC [42] and in the study, clinical correlation analysis also exhibited higher riskscores were found in female NSCLC patients, consisted with previous publishments. Moreover, high-risk scores were significantly positive-associated with severe clinical stages including general stages and TNM stages, suggesting risk scores were closely related to the poor prognosis of NSCLC.

In addition, high risk-scores were significantly negatively correlated with immune activation responses especially T cells activation through the immune infiltration analysis. The killing effect of CD8+ T cells especially cytotoxic T lymphocytes has been considered as the major effector cells in the anti-tumor process. CD8+ T cells could discriminate particular tumor-associated antigen and destroy cancer cells directly in various cancers, including oesophageal cancers [43], colorectal cancers [44]and gallbladder cancers [45]. Through secreting cytokines and attracting inflammatory cells to tumor cells, such as macrophages, neutrophils and NK cells, CD4+ T cells played an essential role in orchestrating the immune responses to cancers [46]. Hiraoka’s study also demonstrated the concurrent infiltration of CD8+ and CD4+ T cells was a favorable prognostic factor in NSCLC [47]. All these studies indicated the loss of CD4+ and CD8+ T cells might lead to the poor prognosis in high-risk NSCLC groups. Interestingly, although the high-risk groups were associated with the poor prognosis, the expression of immune check points was obviously elevated in the patients with high risk-scores, implying those patients might be sensitive to the immunotherapy, such as *PD-1/PD-L1* inhibitors.

To further construct effective models for predicting the prognosis of NSCLC, we combined the risk prognostic signatures with other clinical characteristics based on multivariate Cox regression analysis. To better predict the one/three/five years survival of NSCLC for each individual, we successfully established the nomogram by incorporating age, gender, TNM stages and risk scores. Calibration curves exhibited that the nomogram had good prediction capacity in both three-year and five-year overall survival and its clinical practicability was also validated in DCA. Besides, data from three GEO dataset also confirmed that high-risk groups were associated with worse overall survival than low-risk groups with excellent AUC value. In the validation datasets, high-risk subgroups were also positive associated with female and high clinical stages and also discriminated NSCLC patients with poor outcomes. Furthermore, ROC curves showed that risk scores could be used to predict the prognosis for NSCLC combined with traditional clinical indices, with a high mean AUC value of 0.784. The results of our analysis using a ranking method with an RF model showed that risk-score was the most significant index for the prognosis of NSCLC, with greater mean decrease of Gini and Accuracy than other clinical indexes. These findings indicated that we might be able to evaluate and predict the prognosis of NSCLC through measuring the expression levels of the risk signatures to infer the risk scores.

Furthermore, to validate the significance of risk-scores in the prediction of immunotherapy, the patients receiving anti-PDL1 immunotherapy were evaluated based on external datasets and we found the risk scores were significantly higher in patients responded to corresponding immunotherapy, suggesting target immunotherapy might be beneficial tool for the patients with high risk-scores. Besides immunotherapy, common chemotherapeutic drugs were also be demonstrated lower IC50 value in high risk-score cohorts, implying the high risk-score patients might be more efficacious against these chemotherapeutic drugs. Overall, these findings from external datasets validated the potential benefits in high risk-scores and indicated risk scores might play a vital role in predicting the curative responses to common chemotherapy and immune checkpoint therapy.

However, there are still several limitations in our study. For one thing, although the risk prediction model for the prognosis of NSCLC was proposed and validated by TCGA and GEO datasets, the accuracy and clinical application of this model was still need more external congeneric researches, even clinical practices, to repeatedly confirm and improve. In addition, our study only found the association between the risk scores and poor prognosis in NSCLC while the detailed role of these risk genes in the pathogenesis of NSCLC remains to be further verified by in-depth in vivo and in vitro studies.

## Conclusion

In conclusion, our study firstly proposed the immune molecular subtypes based on clustering immune-cell infiltration scores with distinct clinical and immunological signatures in both LUAD and LUSC patients. Moreover, we identified and validated the immune risk prognostic model combined risk scores and clinical signatures, which can be used as an effective tool to predict the overall survival and immunotherapy efficacy of NSCLC. The various transcriptomic analysis helps us screen significant genetic signatures of NSCLC and provides a new clinical application in predicting prognosis and benefits of immunotherapy for NSCLC.

## Supplementary Information

**Additional file 1: Fig. 1.** The summary and description of the study workflow.

**Additional file 2: Fig. 2.** Comparison of genomic alterations between SubA and SubB in the TCGA datasets. **A**, **B** Differential somatic mutation analysis of the two subgroups in LUSC patients. **C**, **D** Differential somatic mutation analysis between the two subtypes in LUAD patients.

**Additional file 3: Fig. 3.** **A**, **B** Comparison of clinical phenotypes between two subtypes without significant statistical differences in LUAD and LUSC; **C** Comparison of other immune cells between high and low risk-score groups; **D** The expression of these risk genes remarkably increased in tumor patients using immunohistochemistry from HPA database. **E** Comparison of IC50 value between high and low risk-score groups for Paclitaxel and Pemetrexed.

**Additional file 4: Table 1.** Clinical information of external validation datasets of NSCLC.

## Data Availability

Publicly available datasets were analyzed in this study. This data can be found here: Gene Expression Omnibus (GEO) (https://www.ncbi.nlm.nih.gov/geo/) (Accessions: GSE37745, GSE31210, GSE50081, GSE135222 and GSE126044); The Cancer Genome Atlas (TCGA) (https://portal.gdc.cancer.gov/) (TCGA Lung Adenocarcinoma (LUAD) datasets; TCGA Lung Squamous Cell Carcinoma (LUSC) datasets) and Genomics of Drug Sensitivity in Cancer (GDSC) databases (https://www.cancerrxgene.org/).

## Abbreviations

NSCLC: Non-small cell lung cancer
LUSC: Lung squamous cell carcinoma
LUAD: Lung adenocarcinoma
DEGs: Different expression genes
TME: Tumor microenvironment
TCGA: The Cancer Genome Atlas
GEO: Gene Expression Omnibus
HPA: Human Protein Atlas
GO: Gene Ontology
FC: Fold change
NES: Normalized enrichment score
ROC: Receiver operating characteristic curves
RF: Random forest
KEGG: Kyoto Encyclopedia of Genes and Genomes
