# Supplementary figures and images for: Prognostic characterization of immune molecular subtypes in non-small cell lung cancer to immunotherapy
Source: BMC Pulm Med. 2021 Nov 29;21:389. doi: 10.1186/s12890-021-01765-3 (PMC8628446; doi:10.1186/s12890-021-01765-3)

Supplementary Fig.1

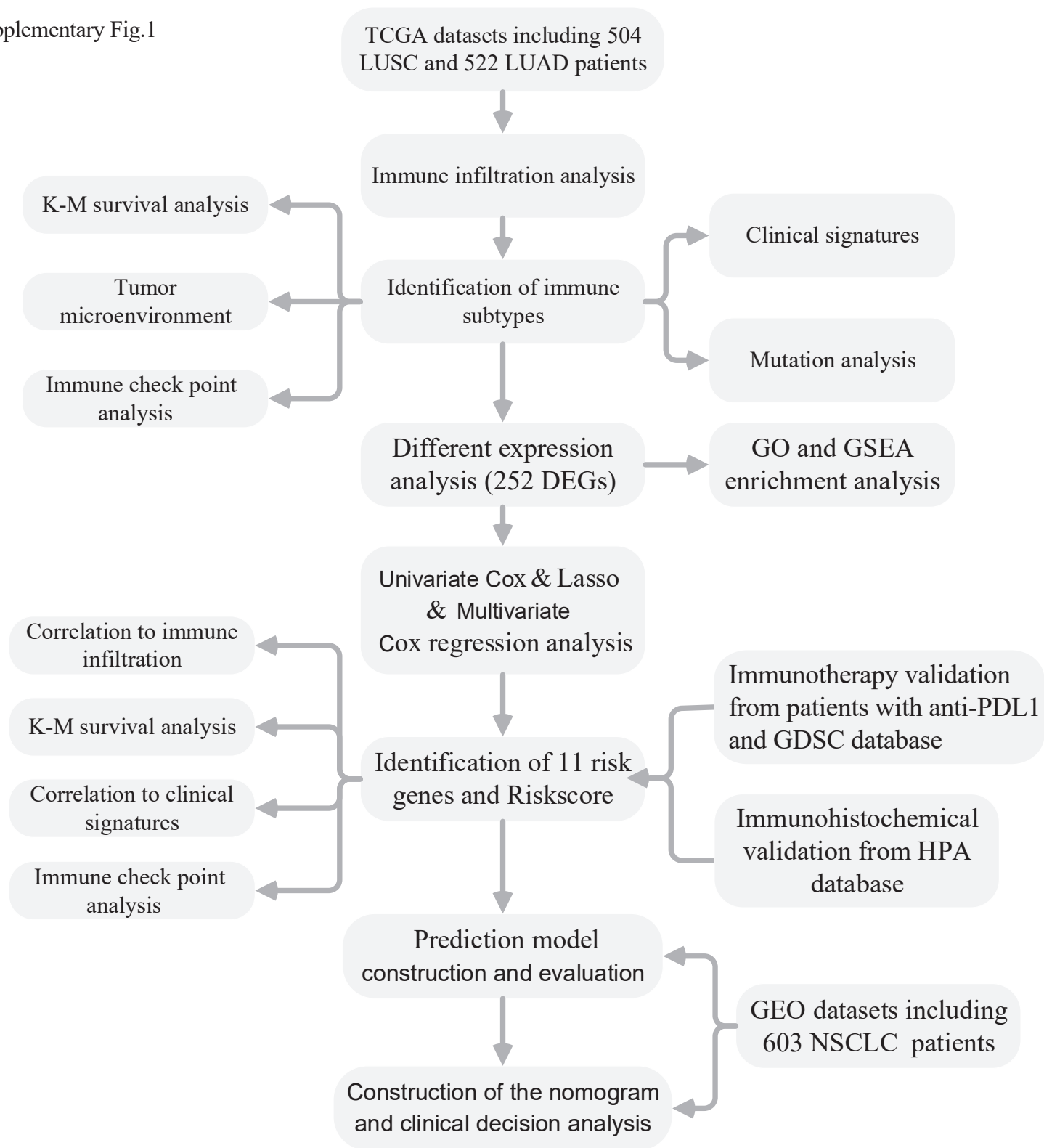

Supplement: Supplementary file 1 — Additional file 1: Fig. 1. The summary and description of the study workflow. [file 12890_2021_1765_MOESM1_ESM.pdf]

Supplementary Figure3

A LUAD

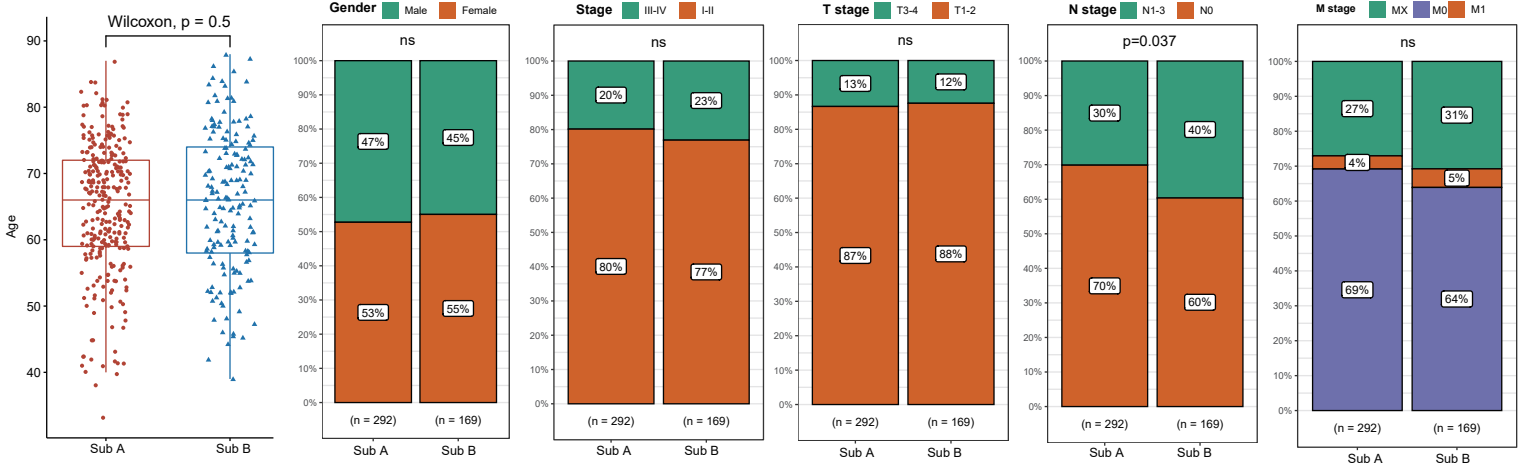

B LUSC

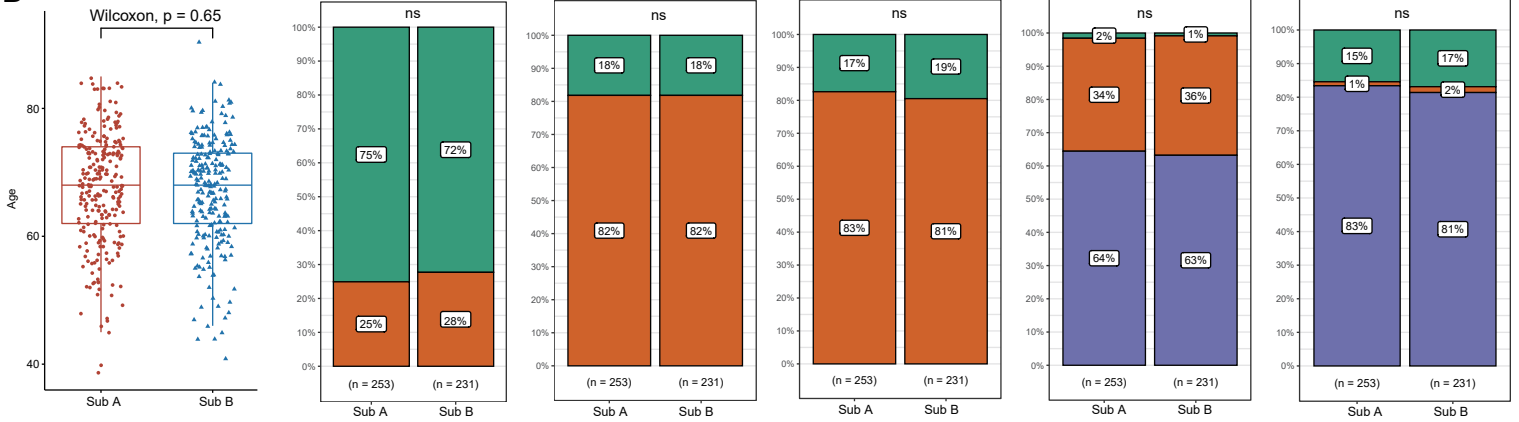

C

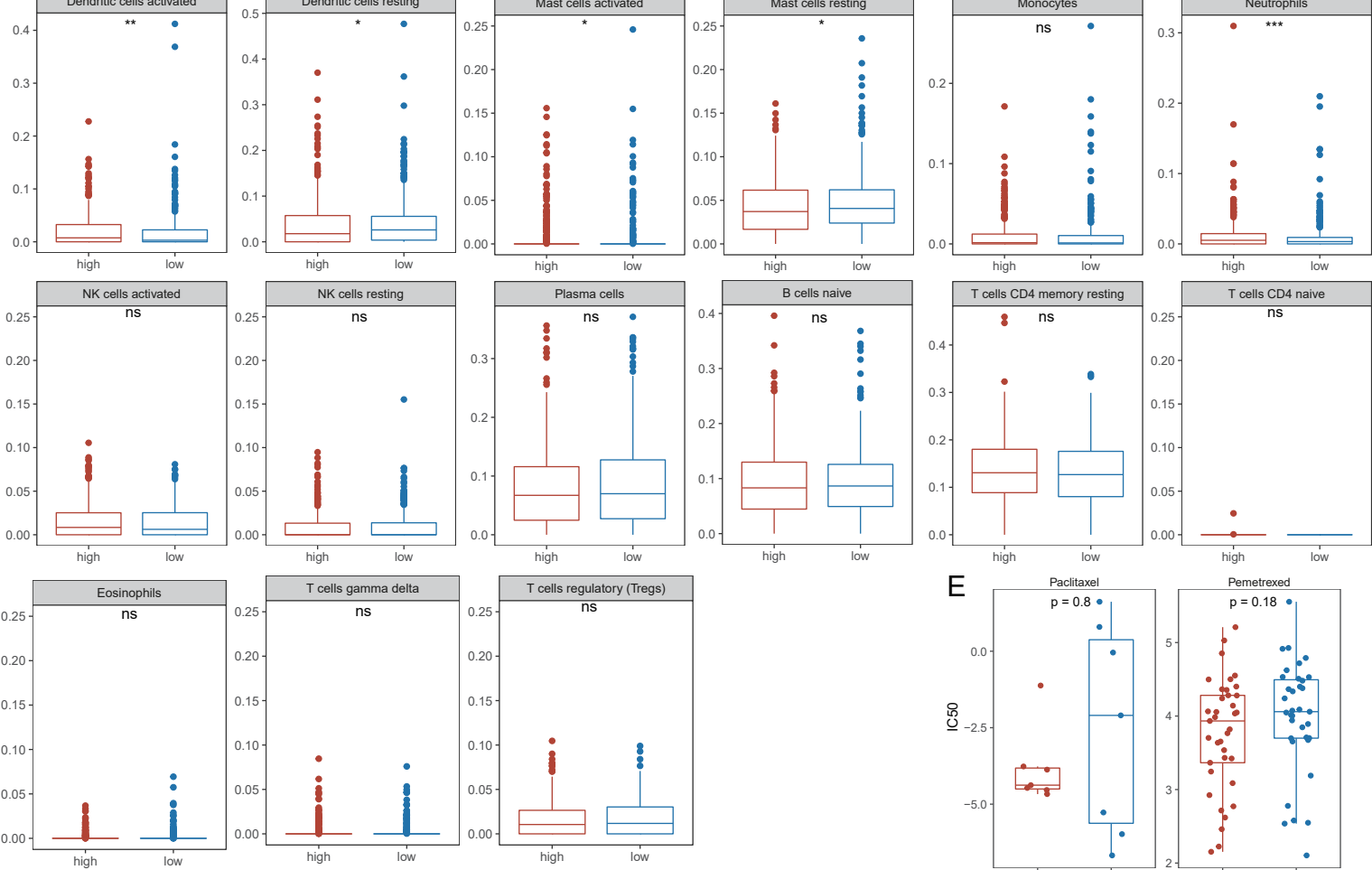

E

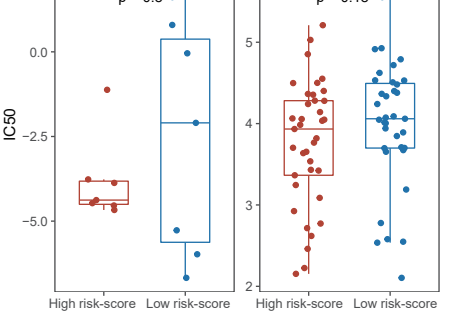

D

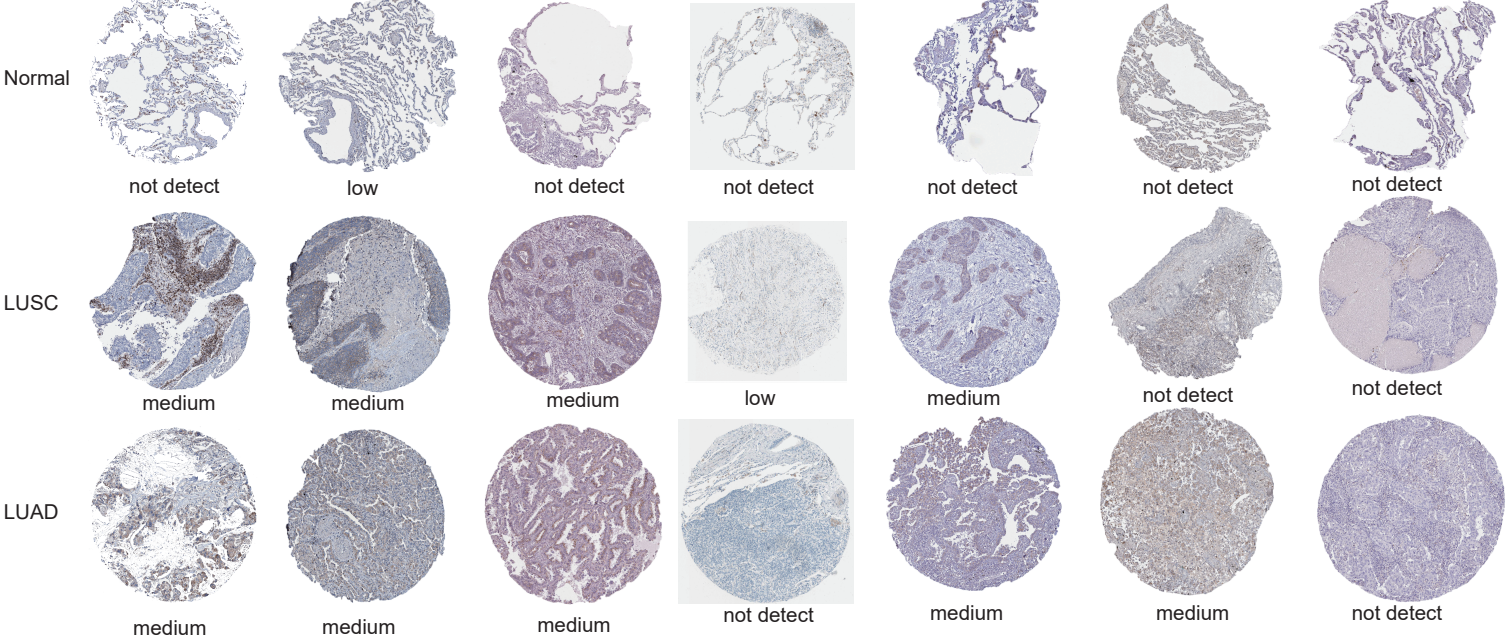

Supplement: Supplementary file 3 — Additional file 3: Fig. 3. A, B Comparison of clinical phenotypes between two subtypes without significant statistical differences in LUAD and LUSC; C Comparison of other immune cells between high and low risk-score groups; D The expression of these risk genes remarkably increased in tumor patients using immunohistochemistry from HPA database. E Comparison of IC50 value between high and low risk-score groups for Paclitaxel and Pemetrexed. [file 12890_2021_1765_MOESM3_ESM.pdf]
