# Supplementary material for: Prognostic characterization of immune molecular subtypes in non-small cell lung cancer to immunotherapy
Source: BMC Pulm Med. 2021 Nov 29;21:389. doi: 10.1186/s12890-021-01765-3 (PMC8628446; doi:10.1186/s12890-021-01765-3)

Supplementary Figure2

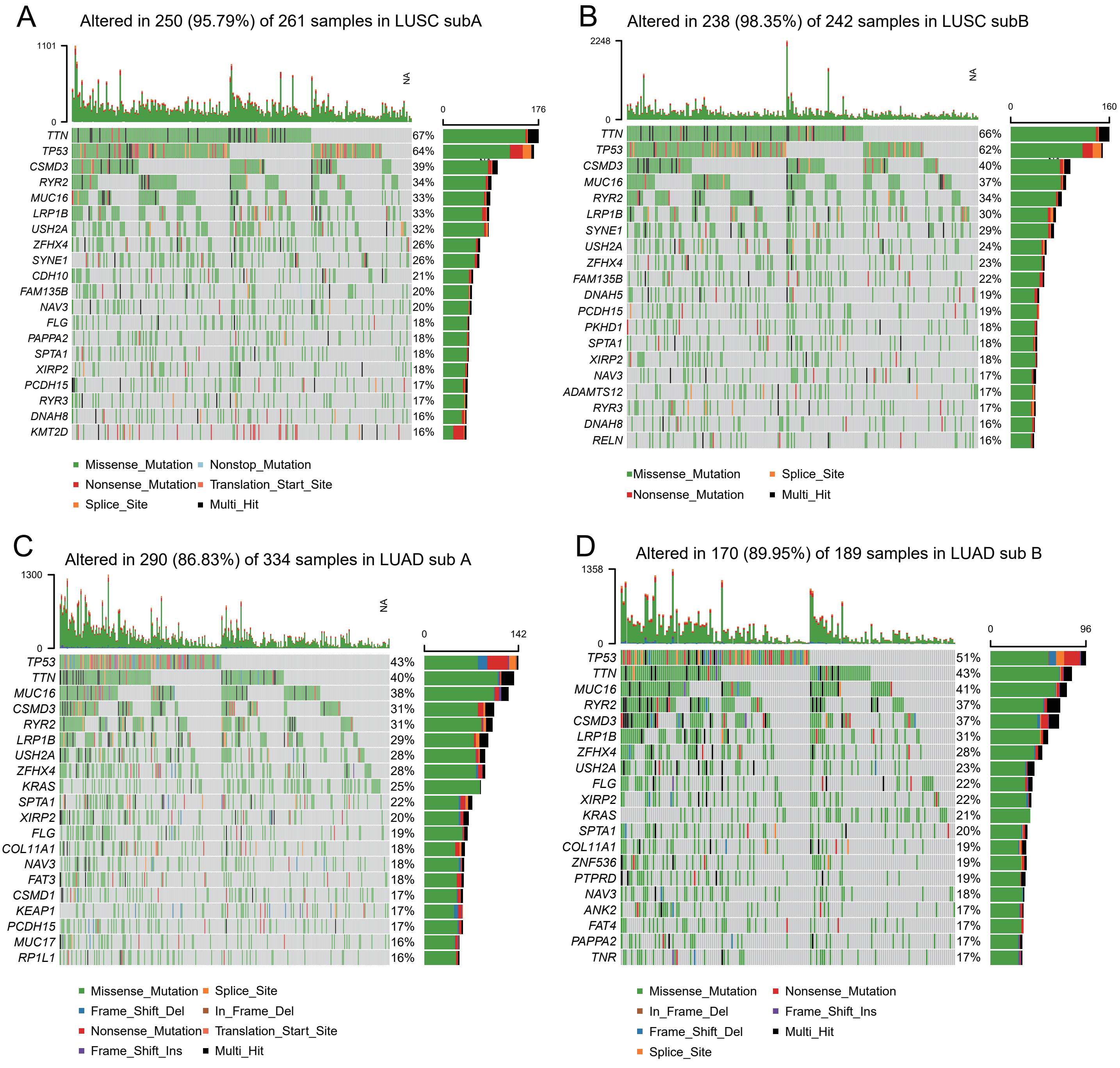

Supplement: Supplementary file 2 — Additional file 2: Fig. 2. Comparison of genomic alterations between SubA and SubB in the TCGA datasets. A, B Differential somatic mutation analysis of the two subgroups in LUSC patients. C, D Differential somatic mutation analysis between the two subtypes in LUAD patients. [file 12890_2021_1765_MOESM2_ESM.pdf]
