# Supplementary material for: Prognostic characterization of immune molecular subtypes in non-small cell lung cancer to immunotherapy
Source: BMC Pulm Med. 2021 Nov 29;21:389. doi: 10.1186/s12890-021-01765-3 (PMC8628446; doi:10.1186/s12890-021-01765-3)
Supplement: Supplementary file 4 — Additional file 4: Table 1. Clinical information of external validation datasets of NSCLC. [file 12890_2021_1765_MOESM4_ESM.docx]

**Supplementary Table1. Clinical information of test datasets of NSCLC**

| **Characteristics** | **GSE37745(196)** | |  | **GSE31210(226)** | |  | **GSE50081(181)** | |
| --- | --- | --- | --- | --- | --- | --- | --- | --- |
|  | **number** | **percentages (%)** |  | **number** | **percentages (%)** |  | **number** | **percentages (%)** |
| **Tumor type** |  |  |  |  |  |  |  |  |
| **NSCLC** | 196 | 100.00% |  | 226 | 100.00% |  | 181 | 100.00% |
| **LUAD** | 106 | 54.08% |  | - | - |  | 128 | 70.72% |
| **LUSC** | 66 | 33.67% |  | - | - |  | 42 | 23.20% |
| **LCLC** | 24 | 12.24% |  | - | - |  | 8 | 4.42% |
| **ASC** | - | - |  | - | - |  | 3 | 1.66% |
| **Age** |  |  |  |  |  |  |  |  |
| **<65** | 94 | 47.96% |  | 163 | 72.12% |  | 59 | 32.60% |
| **≥65** | 102 | 52.04% |  | 63 | 27.88% |  | 122 | 67.40% |
| **Gender** |  |  |  |  |  |  |  |  |
| **male** | 107 | 54.59% |  | 105 | 46.46% |  | 98 | 54.14% |
| **female** | 89 | 45.41% |  | 121 | 53.54% |  | 83 | 45.86% |
| **Survival status** |  |  |  |  |  |  |  |  |
| **alive** | 51 | 26.02% |  | 163 | 72.12% |  | 106 | 58.56% |
| **dead** | 145 | 73.98% |  | 63 | 27.88% |  | 75 | 41.44% |
| **Survival time/days** | 1762.31±1493.73 | - |  | 1722.92±687.72 | - |  | 1548.87±863.08 | - |
| **Clinical Stage** |  |  |  |  |  |  |  |  |
| **Stage I-II** | 165 | 84.18% |  | 226 | 100.00% |  | 181 | 100% |
| **Stage III-IV** | 31 | 15.82% |  | 0 | 0.00% |  | 0 | 0.00% |
| **T stage** |  |  |  |  |  |  |  |  |
| **I-II** | - | - |  | - | - |  | 179 | 98.90% |
| **III-IV** | - | - |  | - | - |  | 2 | 1.10% |
| **N stage** |  |  |  |  |  |  |  |  |
| **N0** | - | - |  | - | - |  | 129 | 71.27% |
| **N1-3** | - | - |  | - | - |  | 52 | 28.73% |
| **M stage** |  |  |  |  |  |  |  |  |
| **M0** | - | - |  | - | - |  | 181 | 100.00% |
| **M1** | - | - |  | - | - |  | 0 | 0.00% |

Note: NSCLC, non-small cell lung cancer; LUAD, lung adenocarcinoma; LUSC, lung squamous cell carcinoma; LCLC, large cell lung cancer; ACS, adenosquamous carcinoma.
